# The California Managed Care Organization Tax and Medi-Cal Patients in the Emergency Department

**DOI:** 10.5811/westjem.35257

**Published:** 2024-09-19

**Authors:** Lauren Murphy, Gita Golonzka, Ellen Shank, Jorge Fernandez

**Affiliations:** * California State University, Chico, Chico, California; † California Chapter of the American College of Emergency Physicians, Sacramento, California; ‡ University of California San Diego, School of Medicine, La Jolla, California; § University of California Davis Health, Department of Emergency Medicine, Sacramento, California

Last week, Timothy^*^, a 60-year-old unhoused gentleman, presented to the ED requesting medical clearance. When asked why, he explained that he was staying at a warming center and had been separated from his wife and quarantined in a separate sleeping space due to concerns that he was infectious. He then doffed his beanie cap to reveal a 10 × 10-centimeter fungating squamous cell carcinoma that had been thriving on his scalp for the past two years. He had moved counties a few years back and since then had had difficulty reestablishing and understanding his coverage within a geographically managed care system. He was on the waiting list to see a dermatologist who accepted Medi-Cal (California’s version of US Medicaid health insurance for the poor). Further chart review revealed that, following at least three ED visits, he had been referred to health navigators to try to secure a dermatologist appointment; but he had been waiting over a year and now was simply requesting medical clearance to be reunited with his wife. This case was a stark example of the arduous barriers some patients in California must overcome to receive care and the hurdles that emergency physicians (EP) must surmount to help these covered patients access the follow-up care they require.

## History of the Managed Care Organization (MCO) Tax

Medi-Cal is funded through state and federal dollars. The federal government uses the Federal Medicaid Matching Rate to calculate how to match state spending on Medicaid programs. For every dollar spent on Medicaid, a state can receive at least $1 in Medicaid federal financial participation (FFP) funding. Because many states struggled to generate enough revenue to cover their share of Medicaid costs, in 1985 the Health Care Financing Administration allowed states to accept donations from private medical care providers and deemed these donations eligible for FFP matching.^1^ In 2006, Congress gave states the authority to tax providers, including managed care organizations (MCO), to meet their share of Medicaid spending.^2^ According to the Medicaid and Children’s Health Insurance Program Payment and Access Commission, 49 states used some form of provider tax to fund their Medicaid programs in 2019.^3^

California has used an MCO tax for over 20 years to receive federal FFP matching funds. The tax must be authorized by the legislature and is subject to federal approval. The authorizing legislation includes an end date for the tax, requiring it to be reauthorized periodically. Historically, California has used the monies generated by FFP dollars to backfill the General Fund deficit or to fund an array of public services and systems outside the Medi-Cal program.^4^

## 2023 Managed Care Tax Agreement

When the authorizing legislation for the previous MCO tax was nearing its sunset date, a coalition of providers and healthcare facilities came together to negotiate a new MCO tax agreement. The proposal by the coalition was to dedicate the additional funds to provide a Medi-Cal rate increase for a variety of healthcare providers. The increase was proposed to roll out over a series of years. In the first fiscal year 2023–24, a subset of primary care clinicians, reproductive health services, and some outpatient mental health services would receive increases effective January 1, 2024. It also proposed to appropriate $1.28 billion for primary care rate increases and $1.15 billion in specialty rate increases effective January 1, 2025.^5^ There were additional funds allocated for facilities and transport, reproductive health, and mental health that would also take effect in 2025. The coalition argued that California’s Medi-Cal reimbursement rates were objectionably low, forcing clinicians to limit the number of Medi-Cal patients they see, thereby limiting patient access to care.

As the Legislature was considering the coalition proposal, the California Chapter of the American College of Emergency Physician (CalACEP) advocacy team lobbied for a specific pool of funding exclusively for EPs, arguing that the funding was necessary due to their exclusion from previous Medi-Cal physician increases and their disproportionate care for the Medi-Cal population relative to other clinicians. As a result of its advocacy, a $200 million state budget line item was included to bring EM Medi-Cal rates to 87.5% of Medicare.^6^

## May Revision and Final Budget

The agreement between the coalition, CalACEP, and the California Legislature was codified in legislation in 2023 and was scheduled to take effect January 1, 2025. However, because the state budget is only a one-year document and the Legislature does not have the sole power to create the state’s budget, the MCO tax agreement for the 2024–25 fiscal year was subject to negotiation once again between the Legislature and the governor. Governor Newsom included the agreed rate increases in his proposed budget released in January 2024, but his revised budget released in May removed the rate increase entirely and used the MCO tax surplus to backfill California’s General Fund deficit.^7^

The CalACEP advocacy team used a variety of strategies to lobby legislators to restore the $200 million for EP rate increases. Staff lobbied members of the Budget Subcommittees in person for each house of the Legislature to explain the impact of low reimbursement on emergency medicine practice and how it effects access to care for patients. Staff also coordinated a targeted social media campaign that focused on the Sacramento area to keep the issue in the minds of stakeholders. Members of the CalACEP Executive Committee wrote letters to the editors of major news organizations throughout the state. Finally, CalACEP coordinated a grassroots campaign to encourage EPs to contact legislators directly and tell personal stories about their patients and Medi-Cal access. Ultimately, the $200 million for EP rate increases ***were*** included in the budget that Governor Newsom signed on June 29, 2024. However, none of the other previously promised rate increases for other specialties, which had been scheduled to take effect January 1, 2025, were included.

## Proposition 35

While EPs were able to get their increase restored, other physicians were not so fortunate. The California Medical Association, with the support of organizations that were a part of the 2023 MCO coalition, qualified an initiative for the 2024 California State Ballot that, if passed by California voters, would permanently enshrine the agreement reached by the Coalition and the Legislature in 2023 into law. Proposition 35 would require the state to use the MCO tax revenues to fund increases for the Medi-Cal program for the duration of the current MCO tax authorization. This would restore the revoked Medi-Cal rate increases for other clinicians and protect the EP increase for years to come, without having to renegotiate with the Legislature and governor every year, as is typically required by the annual budget process. As of August 2024, the proposition seems to have broad bipartisan support. Numerous healthcare organizations including the California Medical Association, Planned Parenthood, the California Hospital Association, and CalACEP formally support the proposition. Other supporters include the California Hawaii State Conference National Association for the Advancement of Colored People, the Insure the Uninsured Project, the California Democratic Party, and the California Republican Party.^8^

The proposition has no registered opposition, but Governor Newsom has expressed serious concerns in the press about the impact the proposition will have on the ability of the Legislature and governor to deliver a balanced budget in the future.^9^

## Impact on Patients and Clinicians

Emergency departments (ED) are uniquely poised to suffer the strain of insufficient Medi-Cal funding, as patients covered by Medi-Cal seek care in the ED at higher rates than patients with other types of insurance. Since 2019, one of three Californians are covered by Medi-Cal,^10^ but 42% of visits to the ED are by Medi-Cal patients.^11^ This discrepancy is largely driven by low physician participation in Medi-Cal. California’s low Medi-Cal reimbursement rates for physicians result in very few primary care physicians and specialists who accept patients with Medi-Cal and long wait times.^12^ This often leaves the sickest and most vulnerable patients with nowhere to go except the ED. Emergency physicians operate under the Emergency Medical Treatment and Labor Act, enacted in 1986, which ensures that all patients arriving in the ED will receive treatment regardless of insurance status or ability to pay. Given the current state of Medi-Cal funding, EPs are forced to navigate not only direly sick and urgent cases, but to fill gaps for preventive and specialty care. This leaves them scrambling to find and coordinate follow-up care. It also leaves them with the moral injury of showing up to shifts day in and day out without being able to get their patients the transfer and follow-up care they need. This is taking a toll on patients and EPs alike.

While EPs are proud to help patients when they need it most, the current funding conditions are unsustainable. Despite the number of ED patients increasing, underfunded departments are decreasing the number of EPs working, and in some cases are employing more physician assistants and nurse practitioners.^13^ In turn, California faces difficulty in hiring and retaining well-qualified and experienced EPs, particularly in historically under-resourced areas.^14^ These changes impact all ED patients in the form of longer wait times and, in the worst cases, poor patient outcomes.

The impact of Medi-Cal underfunding reverberates far beyond emergency services, especially as Medi-Cal eligibility criteria expands. Under the current funding scheme, an increasing number of patients covered by Medi-Cal in California’s healthcare system will result in deeper inequity, as more Medi-Cal patients are competing for the same few spots from a limited number of physicians. A proportional increase in access to physicians is needed, and this must come in the form of increased funding, such as the Medi-Cal rate increases outlined in Proposition 35.

Emergency physicians need a sufficient network of primary care and specialty physicians willing to accept Medi-Cal to provide comprehensive and timely care to patients. Adequate networks would enable patients like Timothy to receive the care they need, when they need it, as opposed to the current system of backlog and waiting. Increasing reimbursement to more closely match the cost of care, and protecting dedicated funds, will improve efficiency and equity in the healthcare system, ultimately improving the quality of care for all Californians.

## Footnotes

* Patient name has been changed to protect confidentiality.
